# The Influence of Different Kinds of Incentives on Decision-Making and Cognitive Control in Adolescent Development: A Review of Behavioral and Neuroscientific Studies

**DOI:** 10.3389/fpsyg.2018.00768

**Published:** 2018-05-23

**Authors:** Jutta Kray, Hannah Schmitt, Corinna Lorenz, Nicola K. Ferdinand

**Affiliations:** Department of Psychology, Saarland University, Saarbrücken, Germany

**Keywords:** adolescence, incentive type, decision-making, cognitive control, social-emotional system

## Abstract

A number of recent hypothetical models on adolescent development take a dual-systems perspective and propose an imbalance in the maturation of neural systems underlying reward-driven and control-related behavior. In particular, such models suggest that the relative dominance of the early emerging subcortical reward system over the later emerging prefrontal-guided control system leads to higher risk-taking and sensation-seeking behavior in mid-adolescents. Here, we will review recent empirical evidence from behavioral and neuroscientific studies examining interactions between these systems and showing that empirical evidence in support for the view of a higher sensitivity to rewards in mid-adolescents is rather mixed. One possible explanation for this may be the use of different kinds and amounts of incentives across studies. We will therefore include developmental studies comparing the differential influence of primary and secondary incentives, as well as those investigating within the class of secondary incentives the effects of monetary, cognitive, or social incentives. We hypothesized that the value of receiving sweets or sours, winning or losing small or large amounts of money, and being accepted or rejected from a peer group may also changes across development, and thereby might modulate age differences in decision-making and cognitive control. Our review revealed that although developmental studies directly comparing different kinds of incentives are rather scarce, results of various studies rather consistently showed only minor age differences in the impact of incentives on the behavioral level. In tendency, adolescents were more sensitive to higher amounts of incentives and larger uncertainty of receiving them, as well as to social incentives such as the presence of peers observing them. Electrophysiological studies showed that processing efficiency was enhanced during anticipation of incentives and receiving them, irrespective of incentive type. Again, we found no strong evidence for interactions with age across studies. Finally, functional brain imaging studies revealed evidence for overlapping brain regions activated during processing of primary and secondary incentives, as well as social and non-social incentives. Adolescents recruited similar reward-related and control-related brain regions as adults did, but to a different degree. Implications for future research will be discussed.

## Introduction

The development throughout adolescence has received an immense scientific interest in the past decades. Researchers from various disciplines have investigated the typical and atypical development in this period of the lifespan to describe and understand biological, social-emotional, cognitive control, and neurological changes. As a transition phase between childhood and adulthood, adolescence has been considered as a sensitive period with heightened vulnerability and demands for adjustment in behavior (Steinberg, 2005; Crone and Dahl, 2012) and sociocultural processing (e.g., Blakemore and Mills, 2014). A number of significant developmental tasks have to be mastered, such as becoming independent from parents, dealing with dramatic hormonal and physical changes, finding a peer group and close interpersonal relationships, and regulating emotions and feelings. If adolescents fail to solve such developmental tasks, their higher vulnerability may result in major problems of behavioral regulation expressed in delinquent behavior, abnormal substance use, such as binge drinking and drug use, and risky behavior, such as reckless driving, as well as in emotional dysfunctions, such as developing depressions and eating disorders. Scientists and also politicians became sensitive to these problems, as adolescents have a four time higher risk of death as a consequence of accidents, injuries, or suicide than children or adults (cf. Eaton et al., 2008).

Evidence from developmental neuroscience about the interplay between emotional/motivational and cognitive development and brain maturation has strongly inspired new ideas and hypothetical models about changes in brain structure and function and their relation to behavior throughout adolescence. To date, quite a number of comprehensive and excellent reviews addressing this interplay, already exist in the literature (Yurgelun-Todd, 2007; Casey et al., 2008; Steinberg, 2008; Geier and Luna, 2009; Luna et al., 2010; Somerville and Casey, 2010; Somerville et al., 2010; Richards et al., 2013; Crone, 2014; Shulman et al., 2016; for a critical comment, see Van den Bos and Eppinger, 2016). Therefore, we will only briefly summarize the most prominent theoretical conceptions and then highlight the potential advantages of applying neuroscientific methods for providing empirical support of differential functions of incentives (rewards and punishments) on decision-making and cognitive control behavior. In particular, we will focus on the questions whether different kinds and amounts of incentives are processed similarly, have a similar impact on control behavior, and have the same function and importance throughout adolescence. Therefore, we will summarize recent evidence on the influence of primary incentives (e.g., food, liquids, etc.) and secondary incentives (e.g., monetary, cognitive, and social) on decision-making and cognitive control functioning. Given that empirical findings of higher risk taking and reward sensitivity in adolescents seem rather mixed, we have the working hypothesis that the type of incentive may explain the inconsistent findings in the literature. To date, it is relatively unknown whether the subjective value of incentives will change in the transition from childhood to adulthood, and if so, how this might influence current theoretical models and interpretation of research findings. Because our main interest is on developmental changes in processing incentives, we will include only studies investigating a relatively large age range around adolescence and studies comparing at least two age groups, thereby one group of children or adolescents.

## Theoretical views on the interplay between the development of social-emotional and cognitive control processing

Researchers from the field of developmental cognitive neuroscience have suggested that a differential maturation of two brain systems associated with socio-emotional and cognitive control processes can explain the higher reward sensitivity, impulsivity, and risk-taking behavior in adolescence. These so-called dual-system models propose that the social-emotional system including the striatum, medial and orbital prefrontal cortices matures earlier than the cognitive control system including the lateral prefrontal, lateral parietal, and anterior cingulate cortices. According to these models, risk-taking behavior is primarily increased in mid-adolescence as the socio-emotional system is highly activated by incentive-related information whereas the cognitive control system is not yet efficiently developed to regulate this bottom-up driven behavior (e.g., Casey et al., 2008; Steinberg, 2008; Luna and Wright, 2016). Although these models vary in their specific assumptions about the developmental course in these two brain systems, they all agree on a differential maturation of these two brain systems as a source of higher impulsivity, sensation seeking, and risky decision-making during adolescence (for a detailed review, Shulman et al., 2016). The triadic model is the only one that posits three interacting subsystems (Ernst and Fudge, 2009; Ernst, 2014). This model builds upon dual-system models but assumes a third brain system (mainly the amygdala) recruited for processing the intensity of emotions and avoidance behavior.

Clear empirical support in favor for the one or the other model is currently lacking. Most studies did not measure indicators reflecting the socio-emotional and cognitive control brain systems, as well as risky decision-making in common across a wider age range, which makes it difficult or impossible to test the theoretical assumptions of different dual-system models against each other. Moreover, the existing empirical evidence on whether incentives either enhance or hamper decision-making and cognitive control functioning and more so for adolescents than for both children and adults is rather inconsistent. Several reasons might explain these inconsistencies. First, studies vary a lot in the investigated age ranges and most studies only included two age groups to examine age differences (i.e., non-linear age trends cannot be determined). Second, studies also vary in the type of tasks and experimental paradigms applied to measure cognitive control processes in decision-making situations (Richards et al., 2013). Third, the impact of incentives has been investigated with different methods, ranging from questionnaires and behavioral data to neuroscientific methods [mostly, functional magnetic resonance imaging (fMRI) and electroencephalogram (EEG)]. The major advantage of neuroscientific methods here is that the influence of incentives can be observed in different phases of goal-directed behavior, such as during the anticipation/preparation, the decision/response selection, and finally during the feedback/evaluation phase. Indeed, there is already evidence that the same type of incentive can result in a hypoactivation or hyperactivation of the same brain system (e.g., the striatum) in adolescents relative to adults, depending on the processing phase (incentive anticipation or response selection; e.g., Geier and Luna, 2009). Hence, the differential functions of incentives for controlling and regulating behavior may also contribute to the inconsistent findings in the literature and need to be considered as well (cf. Richards et al., 2013). Fourth, one aspect that has been largely neglected is the role of the type and amount of incentives. Receiving 5 cents, a sweet, or a smile can have a different subjective value for individuals and the relative preference for specific incentives may change during developmental transitions. Here, we aim to review recent evidence from neuroscientific studies to answer the question of whether similar or different mechanisms and brain systems are at work when different kinds of incentives motivate behavior.

## Differential functions of incentives on decision-making and cognitive control behavior

How goal-directed behavior is motivated is differently conceptualized across research fields in psychology (for a review, see Braver et al., 2014). For the purpose of this review, we will use the term incentive or incentive value as it is used in the reinforcement learning and cognitive neuroscience literature. Stimuli leading to a larger probability that a specific behavior will be shown more often in the future, and leading to more engagement of individuals toward approaching and consuming them, are positive reinforcers or rewards. In contrast, stimuli leading to a larger probability that a specific behavior will be shown less in the future, and leading toward avoiding them, are negative reinforcers or punishments. Primary incentives are innate, such as food, liquids, or sex, and are often used to modify behavior in animals, while secondary incentives are learned, such as monetary, cognitive, or social ones. Both primary and secondary incentives can vary in their amount, magnitude, probability of occurrence, delay, and so on. Whereas the delay of rewards is relatively well examined in infant research, researchers only recently have started to systematically investigate the effects of the amount, magnitude, and probability of incentives on the development of goal-directed behavior and decision-making (Defoe et al., 2015).

Interestingly, recent advances in cognitive neuroscience have identified different neuronal structures that are associated with incentive value coding in separate phases of goal-directed and choice behavior (for a review, Ruff and Fehr, 2014). Dopamineric neurons in the ventral tegmental area and substantia nigra are assumed to code the anticipation of rewards. The discrepancy between an anticipated value and the received outcome value during learning is also encoded in dopamineric neurons and this prediction error signal is used to update the anticipated value of stimuli to optimally learn and adapt the behavior to actual task demands. Changes in the neuronal activity of the orbitofrontal cortex (OFC) have been observed during receipt or consumption of rewards, while the anterior cingulate cortex (ACC), anterior insula and the amygdala are also activated during experiencing pain and receiving punishment. Finally, the ventromedial prefrontal cortex (vmPFC) is recruited during the decision process when anticipated values and response options need to be integrated (for details, see Ruff and Fehr, 2014). Although the types of cognitive processes and associated brain structures will vary along different experimental paradigms and task demands, we will distinguish between phases of anticipating incentives during preparation or response selection and receiving or consuming incentives during the feedback phase. This will help us to identify differential effects of the same incentives as well as similar effects of different incentives in these phases.

In sum, we will report and summarize results from developmental studies that have investigated the impact of primary and secondary incentives on decision-making (e.g., gambling tasks), on cognitive control (e.g., go-nogo tasks or anti-saccade tasks), and on learning from feedback (e.g., reinforcement learning tasks). Our aim is to examine (a) whether different kinds of incentives may have a common or a different function in different stages of motivated behavior, (b) whether the effects are age-invariant or not, and (c) whether similar brain networks are involved in incentive processing across age. Therefore, we include the main findings from behavioral, EEG, and fMRI studies that are briefly summarized in Tables 1–**3**, respectively, along with information about age ranges, type of task and incentive, and processing stage (only in Tables 2, 3). Note that we include only developmental studies in these tables that at least compared two age groups or investigated a broader age range during adolescence.

**Table 1:** Overview of behavioral studies.

**Authors**	**Age groups (age range in years)**	**Task**	**Incentive type**	**Main results**
Galván and McGlennen, 2013	- Adolescents (13–17) - Young adults (23–35)	Passive reward-delivery task	Primary (water, sucrose, salty or no liquid in neutral option)	- No age differences in reaction to water, sucrose, salty and neutral liquid - Higher positive ratings to sucrose than salty liquids in adolescents than adults on a liquid rating scale
Luking et al., 2014	- Children (7–11) - Young adults (22–26)	Gambling task (card guessing game)	Primary (high and low gains, 4 or 2 pieces; high and low losses, 2 or 1 pieces)	- No age differences in win-stay lose-shift strategy - Children reported more overall positive feelings during the task than adults in a post-scan questionnaire
Grose-Fifer et al., 2014	- Adolescents (13–17) - Young adults (23–35)	Gambling task (card guessing game, reward probability 50%)	Monetary (high and low gains, 32–40 Cents; high and low losses, 6–11 Cents)	Both age groups selected high-monetary incentive cards more often than low-monetary incentive cards
May et al., 2004	Children and adolescents (8–18)	Gambling task (card guessing game)	Monetary (neutral trials, no reward; gain trials, 1 Dollar; loss trials, 50 Cents)	No age differences in win-stay lose-shift strategy
Van Duijvenvoorde et al., 2014	- Adolescents (10–16) - Young adults (18–25)	Gambling task (slot machine task, reward probability 33 and 66%)	Monetary (passed trials, no reward; gain and loss trials, ±10 Cents)	Tendency for risky decisions was not related to age, pubertal development, or reward sensitivity
Ernst et al., 2005	- Adolescents (9–17) - Young adults (20–40)	Gambling task (Wheel of Fortune, reward probability 50%)	Monetary (high and low gains, 4 Dollar or 50 Cents; or reward omission)	- Both age groups more satisfied with high than low gains - Adolescents reported more positive feelings than adults in gain trials in a post-scan questionnaire on incentive delivery
Bjork et al., 2010	- Adolescents (12–17) - Adults (22–42)	Monetary Incentive Delay (MID) Task	Monetary (neutral trials, no reward/ loss; high and low gain and loss trials, 50 Cents or 5 Dollar)	Faster responding and higher accuracy with increasing incentives irrespectively of the valence, but no age differences therein
Bjork et al., 2004	- Adolescents (12–17) - Adults (22–28)	Monetary Incentive Delay (MID) Task	Monetary (neutral trials, no reward/loss; high and low gain and loss trials, 20 Cents, 1 Dollar or 5 Dollar)	No effect of reward magnitude or age group on accuracy or reaction times
Galván et al., 2006	- Children (7–11) - Adolescents (13–17) - Young adults (23–29)	Two-choice reaction time task (reward probability 100%)	Monetary (low, medium, and high number of monetary coins)	Faster reaction times to high than medium and low rewards and this effect is most pronounced in adolescents
Cohen et al., 2010	- Children (8–12) - Adolescents (14–19) - Aduts (25–30)	Probabilistic learning task (83% predictable and random condition)	Monetary (no-reward vs. high and low gain trials, 25 or 5 Cents)	Faster responding to large than small incentives only for the adolescent group
Unger et al., 2014	- Children (10–11) - Mid adolescents (13–14) - Late adolescents (15–17)	Reinforcement learning task (100% valid feedback)	Monetary (no-incentive vs. gain and loss trials, 37 Cents)	- Faster responding and better accuracy on win and loss trials for all age groups - Faster learning for older participants but no age differences in interaction with incentives
Santesso et al., 2011	- Adolescents (16–17) - Young adults (18–29)	Gambling task (60:40% win-loss ratio)	Monetary (high and low gains and losses, 195–205 Cents or 45–55 Cents)	- Adolescents and adults do not differ in reward and punishment sensitivity in personality scales and post-experimental questionnaires - Slower response times when two low or high cards were presented compared to one low and one high card
Van Leijenhorst et al., 2006	- Early adolescents (9–12) - Young adults (18–26)	Gambling task (cake task, high and low risk trials)	Cognitive (gain and loss trials; 1 point)	- Both age groups made better predictions under low-risk than high-risk trials and this performance difference was most pronounced in young adolescents
Teslovich et al., 2014	- Adolescents (11–20) - Adults (22–30)	Random Dot Motion Task	Cognitive (high and low gain trials, 5 or 1 points)	Slower responding for large rewards in the group of adolescents relative to adults, who showed slower responding to small rewards
Paulsen et al., 2015	Children and adolescents (10–22)	Inhibitory control (antisaccade task)	Cognitive (no-reward vs. gain and loss trials, 5 points)	- No differences in reaction times between neutral, gain or loss condition - No age differences in incentive processing
Padmanabhan et al., 2011	- Children (8–13) - Adolescents (14–17) - Adults (18–25)	Inhibitory control (antisaccade task)	Cognitive (no incentive vs. potential gain of points)	Adolescents improved inhibitory control with gains to the adults' performance level
Geier and Luna, 2012	- Adolescents (13–17) - Adults (18–29)	Inhibitory control (antisaccade task)	Cognitive (neutral vs. gain and loss trials, 1–5 points)	No age interaction on loss trials but adolescents made more errors on gain trials
Hämmerer et al., 2010	- Children (9–11) - Adolescents (13–14) - Young adults (20–30) - Older adults (65–75)	Probabilistic learning task (65, 75, or 85% positive feedback probability)	Cognitive (gain and loss of feedback points, 10 points)	- Higher variability in decision-making after loss than gain feedback over all age groups - Adolescents and young adults needed less trials to learn correct responses from trial feedback, showed less variability in decision-making and learned more from gains than from losses as compared to younger and older age groups
Chein et al., 2011	- Adolescents (14–18) - Young adults (19–22) - Adults (24–29)	Risk-taking task (Stoplight task)	Social-induced (alone and peer condition: two friends)	Adolescents but not older age-groups exhibited more risk-decisions when being observed by peers
Jones et al., 2014	- Children (8–12) - Adolescents (13–17) - Young adults (18–25)	Social reinforcement learning task (33, 66, and 100% positive feedback probability)	Social-induced (positive and no positive social feedback)	- Independent of age, rare probability of positive feedback led to more false answers than both continuous or frequent positive feedback - Adolescents demonstrated a lower positive learning rate than children and adults - Participants with a higher positive learning rate were more sensitive to feedback probabilities

**Table 2:** Overview of EEG findings.

**Authors**	**Age groups (age range in years)**	**Task**	**Incentive type**	**Phases**	**Main results**
Crowley et al., 2013	- Children (10–12) - Early adolescents (13–14) - Late adolescents (15–17)	Gambling task (Balloon task, reward probability 50%)	Monetary (no-reward vs. gain trials, 10 Cents)	Receiving incentives	- Larger FRN amplitude to neutral than gain trials - Larger FRN amplitude for males than females - Larger FRN for 10–12 and 13–14 year-olds than 15–17 year-olds irrespective of gains and losses - Longer FRN latency for gain than neutral trials - Longer FRN latency for males than females on gain trials - Reduced latency from 10–12 to 15–17 year-olds irrespective of gains and losses
Gonzalez-Gadea et al., 2016	Adolescents (8–15)	Gambling task (high and low advantageous and disadvantageous decks)	Monetary (high and low gains, 2–4 Dollar; and losses, 1–14 Dollar)	Receiving incentives	- Larger FRN amplitude to losses than gains
Grose-Fifer et al., 2014	- Adolescents (13–17) - Young adults (23–35)	Gambling task (Card guessing game, reward probability 50%)	Monetary (high and low gains, 32–40 Cents; high and low losses, 6–11 Cents)	Receiving incentives	- Larger FRN amplitude for losses than gains - Larger FRN amplitude for low than high gains in males - FRN ratio (low gains vs. losses) smaller in adolescent males - Longer FRN latency to losses than gains - Longer FRN latency to high than low outcomes - Longer FRN latency to high gains and losses than to low gains and losses in adolescent males
Santesso et al., 2011	- Adolescents (16–17) - Young adults (18–29)	Gambling task (60 and 40% win-loss ratio)	Monetary (high and low gains and losses, 195-205 Cents and 45–55 Cents)	Receiving incentives	- Larger FRN amplitude for losses than gains - Larger FRN amplitude for low than high gains - FRN amplitude to gains and losses larger for individuals with high score on sensitivity to punishment scales in a personality questionnaire
Unger et al., 2014	- Children (10–11) - Mid adolescents (13–14) - Late adolescents (15–17)	Reinforcement learning task (100% valid feedback)	Monetary (no-incentive vs. gain and loss trials, 37 Cents)	Receiving incentives	- Larger ERN/Ne amplitude for younger and older adolescents than children - Larger ERN/Ne and Pe in incorrect than correct trials - Reduced Pe in late adolescents compared to younger age groups - Larger Pe in gain than neutral and loss trials - No interaction of age and incentive condition in the ERN/Ne amplitude or Pe amplitude
Lukie et al., 2014	- Children (8–13) - Adolescents (14–17) - Young adults (18–23)	Gambling task (virtual maze, reward probability 50%)	Cognitive (reward and non-reward trials in form of fruits)	Receiving incentives	- No age differences in reward positivity - Longer latency for children in reward positivity
Hämmerer et al., 2010	- Children (9–11) - Adolescents (13–14) - Young adults (20–30) - Older adults (65–75)	Probabilistic learning task (65, 75, or 85% positive feedback probability)	Cognitive (gain and loss of feedback points, 10 points)	Receiving incentives	- Children showed largest overall FRN of all age groups - Children and older adults showed smaller differences between FRN after gains and FRN after losses - Younger adults showed larger enhancement of FRN after losses than children

*FRN, Feedback-related negativity; ERN/Ne, Error-related Negativity; Pe, Error Positivity*.

**Table 3:** Overview of fMRI findings.

**Authors**	**Age groups**	**Task**	**Incentive type**	**Phases**	**Main results**
Galván and McGlennen, 2013	- Adolescents (13–17) - Young adults (23–35)	Passive reward-delivery task	Primary (water, sucrose, salty, or no liquid in neutral option)	- Anticipating incentives - Receiving incentives	- No age and condition interactions in the OFC, IFG, insula and caudate - Stronger activation to sugary liquids in adolescents than young adults in the VS - Adolescents show exaggerated striatal activity to aversive salty liquids relative to young adults
Luking et al., 2014	- Children (7–11) - Young adults (22–26)	Gambling task (card guessing game)	Primary (high and low gains, 4 or 2 pieces; high and low losses, 2 or 1 pieces)	Receiving incentives	- Stronger activation in the dorsal/posterior insula after losses in children than in adults - Stronger activation in the anterior insula after losses in adults than in children
May et al., 2004	- Children and adolescents (8–18)	Gambling task (card guessing game)	Monetary (neutral trials, no reward; gain trials, 1 Dollar; loss trials, 50 Cents)	Receiving incentives	- Larger and later peak activations in the striatum and OFC to gains than losses - No age or gender differences in these activations
Van Leijenhorst et al., 2010	- Children (10–12) - Adolescents (14–15) - Young adults (18–23)	Gambling task (slot machine task, reward probability 50 %)	Monetary (neutral and gain trials; 5 Cents)	- Anticipating incentives - Receiving incentives	- Children and adolescents showed larger activation of the anterior insula to potential gain cues / to neutral cues which were more similar to gain cues - Larger striatal activity to reward delivery in adolescents - Young adults showed larger OFC activation to omission of incentives
Van Duijvenvoorde et al., 2014	- Adolescents (10–16) - Young adults (18–25)	Gambling task (slot machine task, reward probability 33 and 66%)	Monetary (passed trials, no reward; gain and loss trials, ±10 Cents)	Receiving incentives	- Larger medial PFC and VS activations to gains than losses - Activation in medial PFC and VS was related to the tendency to choose the risky option - No age differences in these activations - Individual differences in reward sensitivity were related to activation of VS during development
Ernst et al., 2005	- Adolescents (9–17) - Young adults (20–40)	Gambling task (wheel of fortune, reward probability 50%)	Monetary (high and low gains, 4 Dollar or 50 Cents; or reward omission)	Receiving incentives	- Larger nucleus accumbens and bilateral amgydala activation for gain than loss trials - Larger nucleus accumbens activation in adolescents than young adults during reward omission - Larger amygdala activity to incentive omission in young adults than adolescents - Negative emotion correlated with amygdala response to losses in young adults, positive emotions correlated with nucleus accumbens activity in adolescents
Cohen et al., 2010	- Children (8–12) - Adolescents (14–19) - Adults (25–30)	Probabilistic learning task (83% predictable and random condition)	Monetary (no-reward vs. high and low gain trials, 25 or 5 Cents)	- Anticipating incentives - Receiving incentives	- Greater striatal activation with increasing age - Hypersensitive response to unpredicted rewards in striatum and angular gyrus in adolescents as compared to children and adults - Medial PFC was sensitive to reward magnitude, showing a linear increase in sensitivity with increasing age
Bjork et al., 2010	- Adolescents (12–17) - Adults (22–42)	Monetary Incentive Delay (MID) Task	Monetary (neutral trials, no reward/loss; high and low gain and loss trials, 50 Cents or 5 Dollar)	- Anticipating incentives - Receiving incentives	- Reduced activation in the nucleus accumbens for gain than neutral trials in adolescents relative to adults - No age differences in brain activations
Bjork et al., 2004	- Adolescents (12–17) - Adults (22–28)	Monetary Incentive Delay (MID) Task	Monetary (neutral trials, no reward/ loss; high and low gain and loss trials, 20 Cents, 1 Dollar or 5 Dollar)	- Anticipating incentives - Receiving incentives	- Reduced activation in the VS and amygdala for gain than neutral trials in adolescents relative to adults - No age differences in brain activations
Galván et al., 2006	- Children (7–11) - Adolescents (13–17) - Young adults (23–29)	Two-choice reaction time task (reward probability 100%)	Monetary (low, medium, and high number of monetary coins)	Both anticipating and receiving incentives	- Stronger activation in the nucleus accumbens and lateral OFC with increasing incentives - Adolescents showed larger activation in reward-related brain regions relative to children and young adults
Van Leijenhorst et al., 2006	- Early adolescents (9–12) - Young adults (18–26)	Gambling task (cake task, high and low risk trials)	Cognitive (gain and loss trials; 1 point)	- Anticipating incentives - Receiving incentives	- Higher activation in the OFC and DLPFC for high- than low-risk trials, but no age differences - Larger ACC activation in adolescents on high- than low- risk trials relative to young adults - Both age groups showed a larger activation for receiving negative than positive incentives in the VLPFC - Stronger activation in the OFC for negative vs. positive feedback in early adolescents relative to adults
Teslovich et al., 2014	- Adolescents (11–20) - Adults (22–30)	Random Dot Motion Task	Cognitive (high and low gain trials; 5 or 1 points)	Receiving incentives	- Larger VS activation for larger than smaller incentives for both age groups - Stronger activation in the DLPFC and IPS for adolescents relative to adults when incentives are large
Paulsen et al., 2015	- Adolescents (10–22)	Inhibitory control (antisaccade task)	Cognitive (no-reward vs. gain and loss trials, 5 points)	Receiving incentives	- No age differences in VS activation - Striatal activation was associated with better inhibitory control in neutral trials - Activation in the VS on no-incentive trials was associated with better inhibitory control, especially in adolescents < 17 years, whereas these activations dampened performance for adolescents > 17 years - Negative correlation between age and activation of the amygdala in loss trials
Padmanabhan et al., 2011	- Children (8–13) - Adolescents (14–17) - Adults (18–25)	Inhibitory control (antisaccade task)	Cognitive (no incentive vs. potential gain of points)	Receiving incentives	- Adolescent-specific enhanced striatal activity, associated with reward processing, and enhanced activity in areas responsible for inhibitory control during reward trials
Chein et al., 2011	- Adolescents (14–18) - Young adults (19–22) - Adults (24–29)	Risk-taking task (Stoplight Task)	Social-induced (alone and peer condition: two friends)	Anticipating incentives	- Stronger activation of reward-related brain areas (VS, OFC) during risky decision making in adolescents when peers were watching - Independent of social context, adults engaged lateral PFC more strongly than adolescents - Activity in VS and OFC was associated with risky-decision making in adolescents only
Smith et al., 2015	- Adolescents (14–19) - Adults (24–32)	Decision making (guessing task without risk)	Social-induced (alone and peer condition: two friends)	Receiving incentives	- Stronger activation in the VS in adolescents during decision making when peers were watching
Gunther Moor et al., 2010	- Pre-pubertal children (8–10) - Early adolescents (12–14) - Older adolescents (16–17) - Young adults (19–25)	Feedback processing (social judgment task)	Social-induced (feedback whether a person would like them or not)	- Anticipating incentives - Receiving incentives	- Stronger activation of ventromedial PFC and striatum during the expectation to be liked in older adolescents and adults - Similar activation in ventromedial PFC and striatum in all age groups when expectation to be liked was followed by social acceptance feedback - Linear increase in activation with age in striatum, subcallosal cortex, paracingulate cortex, lateral PFC and OFC when expectation not to be liked was followed by negative social feedback
Jones et al., 2014	- Children (8–12) - Adolescents (13–17) - Young adults (18–25)	Social reinforcement learning task (33, 66, and 100% positive feedback probability)	Social-induced (positive and no positive social feedback)	Receiving incentives	- Anterior to mid insula activation was correlated with the positive prediction error in adolescents - Adolescents engaged putamen and supplementary motor area more than children or adults in response to positive reinforcement - VS and medial PFC equally engaged across age

## How do different incentives influence decision-making and cognitive control?

### Primary incentives

Primary incentives have mainly been applied in animal research to motivate behavioral changes and learning (cf. Schultz et al., 1997). In contrast, rather few developmental studies have investigated the impact of primary incentives on goal-directed behavior and decision-making. In comparison to secondary incentives, primary incentives can be delivered immediately, and therefore may be more valuable, motivating, and salient in children than in adolescents or adults (cf. Luking et al., 2014).

We found three studies that have examined the influence of primary rewards on decision-making (Hayden and Platt, 2009; Galván and McGlennen, 2013; Luking et al., 2014). For instance, Luking et al. (2014) were interested in whether receiving or losing candies modulates behavioral choices. Children and young adults were more likely to repeat the same choice after receiving a candy than after losing one, known as “win-stay—lose-shift” strategy. In a post-experimental questionnaire, children reported more overall positive feelings during the task, suggesting a higher subjective value of sweet incentives for children than for adults. A similar finding has been reported by Galván and McGlennen (2013), who compared the effects of appetitive (i.e., sugary) and aversive (i.e., salty) liquids between adolescents and young adults. Both groups reported positive feelings toward appetitive (i.e., sugary) and negative feelings to aversive (i.e., salty) liquids, and this difference was even more pronounced for adolescents than for adults. Hence, both studies support the view that primary incentives are particularly salient to children and adolescents when compared to adults. A third study investigated only younger adults but considered individual differences in risk taking which is often higher in adolescents (Hayden and Platt, 2009). This study directly compared primary and secondary incentives (sugary liquid vs. money) within subjects. The results indicated that although there were individual differences in either preferring or avoiding risks, these were independent of the kinds of incentives given.

Neuroscientific methods like fMRI are suitable to investigate whether age differences in brain activity occur during the processing of primary incentives, i.e., during anticipating or consuming those (Geier and Luna, 2009; Galván and McGlennen, 2013; Luking et al., 2014). For instance, Galván and McGlennen (2013) found no age differences during the anticipation of positive and negative primary incentives in the ventral striatum (VS), OFC, insula, and inferior frontal gyrus. In contrast, during consumption, they found larger activations in the VS in adolescents than young adults, and this activation was positively correlated with increasingly positive ratings for appetitive sugary liquids in adolescents, but not in adults. However, substantial developmental differences in reward delivery have been detected particularly for aversive primary incentives and the omission of rewards. Here, adolescents relative to adults showed exaggerated striatal responses to the delivery of aversive salty liquids (Galván and McGlennen, 2013), and children had a larger activation in the dorsal/posterior insula after candy losses than adults (Luking et al., 2014).

Taken together, primary incentives seem particularly salient in childhood and adolescence as revealed by self-reports, but had no influence on the behavioral choices itself. On the neural level, adolescents relative to adults showed an increased sensitivity in the VS only during consummatory, but not during anticipatory incentive processing. This pattern of results may support a bias in decision-making in adolescents in a way that behavior is less motivated by potentially rewarding activities but is more tuned toward consumption of risk-related rewards, such as alcoholic drinks, drugs, and future choices (Bjork et al., 2010). More importantly, when carefully controlling for the separation between incentive anticipation and delivery as well as for applying child-friendly incentives to equate motivation between age groups, adolescents tend to be highly sensitive to the loss of incentives, suggesting that the striatum codes susceptibility to punishment regimes in adolescence.

### Secondary incentives

Secondary reinforcers are learned by definition and can be characterized as monetary, cognitive, or social (Montague and Berns, 2002). In the following, we will first review empirical studies examining the effects of monetary incentives, before we turn to cognitive and social ones. Within each section, we will first report behavioral findings (see Table 1), and then the neuronal signatures of incentive processing during different stages as measured with even-related potentials (ERPs) and fMRI (see Tables 2, 3).

#### Monetary incentives

Most of the developmental studies to date applied monetary incentives to investigate age-related differences in incentive processing (cf. Bjork et al., 2004, 2010; Galván et al., 2006; Crowley et al., 2013; Gonzalez-Gadea et al., 2016). Although monetary incentives are easily applicable, studies markedly differ (a) in reward magnitude, ranging from a few cents to several euros per trial, (b) in whether monetary feedback is provided in a trial-based or block-wise manner, and (c) in whether wins and losses are presented with equal probability or loss aversion is considered (Santesso et al., 2011; Kujawa et al., 2014). These differences modify the relative “risk” within the decision-making process that subjects may discount on each trial and need to be considered for comparison across different studies. A further major problem for developmental studies is to compare a fixed amount of money across age groups, as receiving, for instance, 50 cents has a different meaning for children and late-adolescents.

##### Behavioral findings

Studies that have used gambling tasks to examine the impact of monetary incentives on age differences in decision-making often found that choice behavior was age-invariant to the magnitude of monetary incentives (Grose-Fifer et al., 2014) and of risk (Van Duijvenvoorde et al., 2014). For instance, Grose-Fifer et al. (2014) applied a card-gambling task in which monetary wins and losses were either small or large (for details, see Table 1). On each trial, adolescents and adults were to choose either a high- or a low-monetary incentive card. Both age groups did not differ in choice behavior and selected high-monetary incentive cards more often than low-monetary incentive cards. Likewise, Van Duijvenvoorde et al. (2014) used a slot-machine task and compared adolescents and adults in risk taking by manipulating the chance to win (66 vs. 33%) or to lose 10 cents. Again, both age groups did not differ in their choices to play and by this in risk taking (Van Duijvenvoorde et al., 2014). However, monetary incentives were probably too low to induce risky decisions in the later study. May et al. (2004) investigated age differences in a two-choice card guessing game in which children and adolescents had to guess whether the hidden number of an upcoming card was greater or less than five. Correct guesses resulted in a gain of one Dollar and incorrect guesses in a loss of 0.5 Dollar, relative to a neutral condition. This ratio was selected to control for loss-aversion in human decision-making (May et al., 2004). Results showed that age did not account for the amount of variability of choosing the same response after a previous reward (i.e., win-stay strategy) or the opposite response after a previous loss (i.e., lose-shift strategy), suggesting that children and adolescents do not differ in choice-behavior when loss-aversion is considered.

In two studies by Bjork et al. (2004, 2010), the effects of magnitude of the monetary incentives was measured by a modified Monetary Incentive Delay (MID) task in which different cues indicated monetary incentives and risks (e.g., win or lose 0, 0.5, 1, or 5$). While the first study did not reveal an effect of incentive magnitude on task performance (Bjork et al., 2004), in the second study adolescents and adults showed faster responding to target stimuli as incentive magnitude increased on both gain and loss trials and again, there were no age differences in these effects (Bjork et al., 2010).

A similar finding has been reported in a study of Unger et al. (2014) who investigated how monetary incentives change performance in a learning task. Results indicated better performance in the two incentivized conditions, that is, children, mid-adolescents, and late adolescents responded faster and more accurately on gain and loss trials relative to the neutral condition (for details, see Table 1). Again, there were no age differences in performance benefits when incentives were provided during learning. However, Galván et al. (2006) applied a learning task in which children, adolescents, and adults had to respond as quickly as possible to a cue that was associated with either a low, medium, or large incentive value. Although all age groups responded faster to large incentives, the RT-difference between incentive values was largest in the group of adolescents. Similarly, Cohen et al. (2010) found that only adolescents responded faster to large than small incentives as compared to children and adults in a probabilistic learning task.

Considering individual differences, personality scales as well as post-experimental questionnaires further revealed that adolescents and adults do not differ in reward and punishment sensitivity (Santesso et al., 2011), as well as in positive feelings related to large compared to small monetary incentives (Ernst, 2014). However, adolescents reported more positive feelings than adults during winning money, but not during reward omission (Ernst et al., 2005). The latter result has been explained by the larger motivational salience of monetary incentives in adolescence than adulthood (e.g., Ernst, 2014).

In sum, the behavioral data mostly show that children, adolescents, and adults do not differ in choice behavior and risk-taking, as all age groups are more likely to select high than low monetary reward trials in gambling tasks. All age groups also respond faster on high than low incentive trials, achieve higher accuracy on incentive trials than on neutral trials, and there were no age differences in win-stay and lose-shift strategies in learning tasks. There is some evidence that adolescents respond faster to large than small monetary incentives, and that they report more positive feelings after receiving monetary incentives than adults do.

##### EEG findings

Most ERP-studies so far have focused on the processing of incentive delivery. A number of studies applied gambling tasks and measured feedback processing, as indexed by the amplitude of the feedback-related negativity (FRN). Researchers found a larger FRN for loss or neutral than for gain trials (Santesso et al., 2011; Crowley et al., 2013; Grose-Fifer et al., 2014; Kujawa et al., 2015; Gonzalez-Gadea et al., 2016) as well as for small than large monetary gains (Santesso et al., 2011), with only small age differences therein. However, differences in FRN amplitudes to monetary incentives may be modulated by individual differences in emotionality, punishment sensitivity, and gender (Crowley et al., 2013; Kujawa et al., 2015). For instance, Santesso et al. (2011) found larger FRN amplitudes to both gains and losses for those individuals that reported higher levels of punishment sensitivity, also irrespective of age. With regard to gender differences, Grose-Fifer et al. (2014) reported that adolescent males showed larger FRN amplitudes to small than large wins, less FRN-differentiation between low gains and losses, as well as delayed FRN latencies to high losses as compared to females. Furthermore, FRN amplitudes in females (adolescents and young adults) were only modulated by the valence (i.e., larger for losses than for gains), suggesting that females may represent incentives only in the two categories positive and negative. In contrast, males seem more sensitive to the value of incentives, and thereby more prone to risk-taking. As most studies did not report age differences, these findings need to be replicated before strong conclusions can be drawn.

Another study focused on the investigation of error processing, as indexed by the error-related negativity (ERN/Ne) and error positivity (Pe), during response execution, when monetary incentives were anticipated. Applying a reinforcement learning task, Unger et al. (2014) showed a larger ERN/Ne for younger (13–14 years) and older adolescents (15–17 years) than for children (10–11 years) but no modulation of the ERN/Ne by monetary incentives, suggesting that adolescents were better able to represent correct and incorrect responses during learning, irrespective of anticipating positive and negative monetary incentives. The Pe, that is often interpreted as subjective evaluation of responses, was also larger for erroneous than correct responses. Moreover, it was also larger for monetary gains than losses and no-incentives, and was reduced for older adolescents relative to the other two age groups. Again, these effects were not modulated by the incentive manipulation. Hence, although the Pe was sensitive to the value of incentives as well as to age, the two factors did not interact with each other.

To summarize, neuronal correlates associated with coding prediction errors clearly indicate that the magnitude and valence of monetary incentives impact processing of feedback delivery, as reflected in a larger FRN to losses than wins and to small than large wins (at least in males), as well as error evaluation, as reflected in a larger Pe to wins than losses. In contrast, error processing (as reflected in the ERN/Ne) during anticipation of monetary incentives was insensitive to the magnitude and valence of monetary incentives. However, although these neuronal correlates are age-sensitive, no interactions of age with the value of monetary incentives were obtained.

##### fMRI findings

Most of the neuroimaging studies also have investigated processing of monetary incentives with variants of gambling tasks. For instance, May et al. (2004) used a card guessing game to investigate children and adolescents between 8 and 18 years when receiving either a positive incentive (i.e., possibility to win 1 Dollar) or a negative incentive (i.e., risk to lose 0.5 Dollar). They found similar brain activations in the striatum and lateral and medial OFC to the delivery of rewards as compared to previous results in adults. Interestingly, the possibility of receiving positive monetary incentives led to larger and later peak activations in the aforementioned brain regions than that of negative incentives, in line with the view that the striatum and OFC are involved in anticipating and encoding the value of incentives. However, no gender and age differences were obtained in this effect. It should be noted that the positive incentive was twice as much as the negative one. Hence, differences in incentive magnitude might have driven the latter effect (May et al., 2004). Nevertheless, Van Duijvenvoorde et al. (2014) found a similar result in adolescents and adults using a so-called slot-machine task. In this study, neuronal responses to feedback delivery after decisions to take a gamble showed larger activation in bilateral VS and medial PFC for gains than losses. In this study, both gains and losses were equivalent (i.e., winning or losing 10 cents). In line with the previous study, they also found no evidence for age differences in reward-related brain activations.

Cohen et al. (2010) examined feedback processing during the delivery of incentives in children, adolescents, and adults in a reinforcement learning task with large (25 cents) and small (5 cents) monetary incentives for correct responses. This condition was contrasted with a non-incentive condition for incorrect responses. In contrast to the findings from the gambling studies reported above, they found that adolescents had a hypersensitive response to unpredicted rewards in the striatum and the angular gyrus as compared to children and adults. Additionally, a region in the medial PFC was sensitive to reward magnitude, but here, a linear increase in sensitivity was found with increasing age. Galván et al. (2006) also found age differences in incentive processing between children, adolescents, and adults in a learning task in which responses to three types of cues were rewarded with high, medium, and large incentives. Across the whole trial, they found an increased activation in the nucleus accumbens and lateral OFC with larger incentive values. In contrast to the aforementioned studies, adolescents showed enhanced incentive-related activity in the nucleus accumbens relative to both children and adults, whereas larger lateral OFC activity was found in children as compared to the two older age groups. These results suggests a different developmental maturation of incentive-related brain regions, as subcortical structures, such as the nucleus accumbens, seem to become disproportionally activated as compared to the later maturing lateral OFC, supporting top-down cognitive control. The difference between studies might be due to different incentive schedules, as the study by Galván et al. (2006) applied a 100% reward probability schedule whereas the previous study did not.

Other studies not only investigated incentive delivery, but also the anticipation and omission of incentives in order to answer the question of whether increased risk-taking in adolescence results from an overestimation of anticipated incentives, from a higher responsiveness to receiving incentives, or both. To this end, Van Leijenhorst et al. (2010) applied the slot-machine task in early and mid-adolescents and young adults. In the incentive anticipation phase, both groups of adolescents showed larger activation in the anterior insula on trials signaling potential gains, but this effect was absent in the group of young adults. In the outcome phase, the two adolescent groups, but not the young adults, also showed larger activations in the striatum during trials signaling incentive delivery. This finding was corroborated by a quadratic age trend of the VS to rewards. In contrast, young adults showed larger activation of the OFC on trials, signaling incentive omission. These findings support the view that middle adolescence is characterized by overactive incentive-related brain regions, especially during reward delivery. Conversely, OFC activations in young adults to the omission of reward may signal the need for increased attention and adjustment of behavior following negative outcomes that is reduced in adolescents (Van Leijenhorst et al., 2010).

In a similar study, Ernst et al. (2005) investigated brain activations specifically to the omission of incentives (i.e., possibility to win either 4 or 0.5 Dollar or nothing) in a wheel-of-fortune task. For both adolescents and young adults, they found larger brain activations for the delivery than the omission of incentives in the bilateral amygdala and the nucleus accumbens. Whereas reductions in neuronal activations to the omission of rewards were encoded in the amygdala in adults, adolescents showed the same activation difference in the nucleus accumbens. Hence, adolescents and adults seem to differ more reliably in response to negative (i.e., omission) than positive monetary incentives. The weaker involvement of the amygdala in response to the omission of incentives may reflect a lower sensitivity to potential harm and less avoidance of negative situations in adolescents, accompanied by a more active reward-related system as reflected by nucleus accumbens activity. This pattern in turn might explain the higher propensity for risk and novelty seeking in adolescents.

Concerning the anticipation and delivery of monetary rewards during cognitive control, the studies by Bjork et al. (2004, 2010) point to a different pattern of age-related differences. In both studies, they applied a MID task in which five different cues indicated monetary incentives and risks (for details, see Table 3). During incentive anticipation, Bjork et al. (2004, 2010) reported reduced nucleus accumbens, VS, and amygdala recruitment by monetary gains relative to no gains in adolescents as compared to adults. In contrast to the previous studies, age differences in incentive-related brain regions were not obtained during the delivery of rewards. Hence, the results suggest that when incentives are bound to individual performance instead of choice behavior, and are measured in separate stages during anticipation and consummation, differential activation patterns in reward-related and control-related brain regions are observed in adolescents (Bjork et al., 2004; for a similar result using a longitudinal design, see Lamm et al., 2014).

Apart from age differences in neuronal correlates of reward anticipation, delivery, and omission, both the study by Van Duijvenvoorde et al. (2014) and Ernst et al. (2005) emphasized the role of individual differences in personality traits and affective states during gambling. In the former study, activations of the VS and medial PFC for play decisions were related to individual differences in scores on the BAS sub-scale Fun-Seeking: Subjects, who were more willing to approach potentially rewarding events in daily-life, showed a larger activation to incentives in the VS and medial PFC (Van Duijvenvoorde et al., 2014). In the latter study, Ernst et al. (2005) showed reduced amygdala responses to omission of incentives to be correlated with self-reported negative affect in adults, whereas adolescents showed correlations between nucleus accumbens activity and positive affect.

Taken together, results on age differences in brain activations in reward-related and control-related regions are mixed, and vary with the magnitude and probability of monetary incentives as well as with the type of task and stage of processing. When incentive values are high and the uncertainty of receiving them is rather low, an imbalance between the highly activated reward region and low activated control regions may lead to more impulsive and risky decision-making in adolescence.

#### Cognitive incentives

Regarding cognitive incentives, one can differentiate between written feedback concerning performance accuracy on the preceding trial (e.g., Kim et al., 2014) and visual feedback indicating points for correct responses that are counted during performing the task and can be exchanged for monetary compensation at the end of the task (e.g., Paulsen et al., 2015). Also, some studies employ abstract feedback symbols (i.e., smiley, circles, or shapes, cf. Bjork et al., 2004; Kujawa et al., 2015) or category members (i.e., fruits, cf. Lukie et al., 2014) whose (often monetary) value is learned beforehand. These types of incentives are used to reduce the potential impact of age differences in the perceived value of, for instance, monetary incentives, that otherwise could lead to age differences in motivated behavior (e.g., Teslovich et al., 2014).

##### Behavioral findings

Van Leijenhorst et al. (2006) applied a gambling task called “cake task” involving high- and low-risk trials. Early adolescents and young adults had to predict choices of the computer and received either one point for a correct prediction that was in accordance with the computer's (random) choice or a loss of one point for an incorrect prediction. Early adolescents and adults made better predictions under low-risk conditions. Under high-risk conditions, early adolescents were in tendency more prone to risk-taking than adults, as they made more incorrect predictions than adults. However, the high-risk condition in that study might also have induced larger response conflict due to higher perceptual demands. Therefore, it is difficult to conclude whether children were indeed more sensitive to risk taking under high-risk conditions (Van Leijenhorst et al., 2006).

As gambling tasks *per se* do not give rise to age-related differences in task performance (Lukie et al., 2014), other studies investigated the role of cognitive incentives in simple and more complex tasks requiring cognitive control. For instance, using a simple perceptual RT task, Teslovich et al. (2014) found that adolescents were in particular sensitive to high positive cognitive incentives. They showed slower response times when large rewards could be lost, while young adults showed a speeded responding under this condition. Hence, age differences occur with larger magnitude of positive incentives (which acquire a negative value when large rewards are lost). Groom et al. (2010) investigated whether cognitive incentives would increase inhibitory performance in adolescents by varying not only the amount but also the valence of cognitive incentives (see Table 1). The high incentive condition enhanced inhibitory control performance, irrespective of the valence of incentives. Thus, positive and negative cognitive incentives, when strictly comparable in task design, are equally appropriate to foster performance in adolescence. Padmanabhan et al. (2011) also found that children and adolescents showed improvements to adults' performance levels in inhibitory control when potential incentives could be received (Padmanabhan et al., 2011). In contrast, Paulsen et al. (2015) applied an anti-saccade task to measure inhibitory control and investigated the impact of positive and negative cognitive incentives (i.e., gain or loss of 5 points). Their results indicated no effect of incentives on task performance, irrespective of age. Moreover, Geier and Luna (2012) even found negative effects of abstract reward cues (indicating trials with potential wins or losses of points, or neutral trials) on inhibitory control. In this anti-saccade study, adolescents committed more errors on gain trials than adults but not on loss trials. Thus, whether cognitive incentives influence inhibitory control may also depend on the type of response or the demands on inhibitory control.

The effect of cognitive incentives has also been investigated in reinforcement learning tasks (Hämmerer et al., 2010), in which participants performed a probabilistic two-choice learning task resulting in gains and losses of feedback points after each trial. Here, in contrast to children and older adults, adolescents and young adults learned faster from feedback and showed less switching of choices, and this difference was more pronounced after a positive than after a negative cognitive incentive. However, the switching of choices was more frequent after a negative incentive in all age groups.

Together, the behavioral results reveal that cognitive incentives can facilitate inhibitory control in adolescent, but not in children, depending on the type of inhibitory task. While behavioral adjustment after negative cognitive incentives is found across all age groups, adolescents' performance in decision-making is driven by response conflict on high-risk tasks (e.g., not receiving a large positive incentive). The latter effect suggests that losses involve emotional processing that is target to profound maturational changes during adolescence (Hämmerer et al., 2010; Paulsen et al., 2015).

##### EEG findings

Only rather few studies have investigated the neuronal signatures of incentive anticipation and delivery. For instance, the study by Groom et al. (2010) compared ERP correlates during response selection during a go-nogo task in which positive and negative cognitive incentives (gaining vs. losing points vs. neutral condition) were compared between blocks, so that the effects of incentives on task preparation cannot be investigated. Adolescents showed a larger N2-amplitude in positive than negative incentive and neutral blocks, indicating an early attentional process toward processing the positive valence of cues. However, this effect did not interact with demands on inhibitory control, that is, the positive valence effect was not different between no-go and go trials. They also showed a larger P3 amplitude in incentive blocks than in neutral blocks, suggesting a higher processing effort on motivated salient conditions. Again, this effect did not interact with inhibitory control demands (for a similar finding in young adults, see Schmitt et al., 2015).

A larger number of studies has focused on error- and feedback-related components (the ERN/Ne and FRN; for a review on developmental changes in these components, see Ferdinand and Kray, 2014) in order to investigate the impact of cognitive incentives on learning and monitoring processes. One study reported larger amplitudes of the ERN/Ne and Pe for errors than correct responses during an inhibitory control task in adolescents (Groom et al., 2013). However, there was no effect of incentive value (i.e., differences between high or low positive or negative cognitive incentives) on ERN/Ne and Pe. In a similar vein, Lukie et al. (2014) found no age differences in processing different cognitive incentives (i.e., symbolic gains and losses) on amplitudes of the FRN. However, the study was a pure gambling task and therefore one cannot assess the impact of reward feedback on ERP measures of cognitive control and reinforcement learning. To investigate this issue, Hämmerer et al. (2010) applied an incentivized probabilistic learning task in more fine-grained age groups. They found that although having the largest FRN amplitudes overall, children showed smaller differences between FRN amplitudes after gains and losses relative to adolescents and young adults. This pattern remained stable even after controlling for baseline FRN size and for changes in the FRN after gain feedback in each age group. The findings suggest that children are less able to yield a differentiated classification of favorable and less favorable outcomes for task-specific goals, as indicated by FRN ratio scores, and to use cognitive feedback for adapting to task-specific goals (for a similar result, see Ferdinand et al., 2016).

##### fMRI findings

Brain imaging studies on developmental changes during anticipating and receiving cognitive incentives have revealed large activation overlap in brain networks between early adolescents and adults (Van Leijenhorst et al., 2006). In particular, Van Leijenhorst and colleagues examined age differences in brain activations during the decision-making process itself and processing feedback between low- and high-risk conditions in selected regions of interest. During decision-making, they found higher activations in the OFC and DLPFC for high- than low-risk conditions but no age effects in this difference, suggesting similar recruitment of brain regions known to be involved in anticipation of incentives and representation of risk options in early adolescents and adults. However, adolescents mainly differed from adults in higher activations of the ACC on high- than low-risk trials, suggesting that they perceived either more conflict or needed to engage more heavily in performance monitoring during high-risk choices. This finding was in line with the behavioral results, showing more incorrect decisions in early adolescents during higher uncertainty for correct predictions (Van Leijenhorst et al., 2006).

The study by Van Leijenhorst et al. (2006) also assessed age differences in receiving cognitive incentives (i.e., during feedback processing). They found that both age groups showed a larger activation for receiving negative than positive feedback in the VLPFC, known to be recruited during punishment. Moreover, early adolescents showed a larger activation in the right lateral OFC for negative than positive cognitive incentives, irrespective of the risk level, while for young adults this difference in brain activation was less pronounced. The age difference was due to differences in brain activations on negative feedback trials, suggesting that early adolescents were more sensitive to negative than positive feedback. This region is associated with coding the magnitude of both positive and negative outcomes and with implementing behavioral adjustments after negative feedback (Tsuchida et al., 2010). However, the study did not manipulate the magnitude of incentives as, for instance, the study by Teslovich et al. (2014). They compared receiving a small (1 point) and a large (5 point) positive incentive between adolescents and adults in three regions of interest, in the VS, IFC, and DLPFC. The results indicated a larger activation in the VS for larger than smaller positive incentives, but no age differences in this effect. In contrast, adolescents showed larger activations in the IPS and DLPFC for larger than smaller rewards. The increased activation of the fronto-pariatal network for higher incentives in adolescents has been interpreted as a bias in response selection in order to slow down responding until enough evidence is accumulated for a correct decision. Unfortunately, this latter study did not separate anticipation and delivery of incentives, and did not manipulate the magnitude of negative incentives so that the results of both studies are difficult to compare.

Padmanabhan et al. (2011) examined the effects of rewards on inhibitory control in an anti-saccade task. They investigated children, adolescents, and adults and compared conditions with an abstract cue indicating a potential win (that was later converted into a monetary bonus) and with an abstract cue that served as a neutral trial. They found an adolescent-specific enhancement in VS activity, and in areas responsible for inhibitory control during reward trials. Paulsen et al. (2015) investigated the contribution of age and inhibitory control performance on fronto-striatal activations in the anti-saccade task in 10–22 year-olds during positive and negative cognitive incentives (gaining vs. losing points vs. neutral). Although striatal activation during the decision-making process on neutral trials was associated with overall better inhibitory control, younger and older subjects did not differ in striatal activation during positive incentive conditions. However, inhibitory control performance in adolescents younger than 17 years benefitted from fronto-striatal activation during neutral trials, whereas these activations hampered anti-saccade performance in adolescents from 17 years on. Interestingly, age was negatively correlated with activation in the amygdala during loss trials only, suggesting that the amygdala of younger adolescents was more sensitive to losses. The results suggest a transition phase of fronto-striatal recruitment in adolescence, in which fronto-striatal regions benefit cognitive control performance and in which emotional processing in the amygdala mediates bottom-up processing during inhibitory control in younger adults (Paulsen et al., 2015).

Together, although ERP and fMRI methods are well suitable to examine whether (cognitive) incentives influence decision-making and cognitive control behavior in different stages, the existing studies have rarely made use of it. From EEG studies, we have learned that during the decision-making processing (anticipation of incentives) both children and adults show enhanced attention and processing effort under motivated conditions as compared to neutral ones, indexed by larger N2 and P3 amplitudes. Children and adults are also similarly sensitive to risky decisions, as they show similar changes in brain activation in prefrontal regions (OFC, DLFPC) when positive incentives are less likely. Here they differ only in higher ACC activation, signaling higher conflict processing in such situations. During response selection and receiving feedback, it seems that children are less able to differentiate between positive and negative cognitive incentives as reflected in smaller FRN difference scores than in adolescents and adults. Both adolescents and adults are sensitive to negative cognitive incentives, indicated by a larger recruitment of the VLPFC on negative than on positive incentive trials. In contrast, adolescents show a larger recruitment of cognitive control networks, and a lower amygdala activation in response to losses.

#### Social incentives

Given that the processing of social information underlies dramatic developmental changes over the course of adolescence, and that the social context might be the most salient factor influencing the behavior of youth (Crone and Dahl, 2012), it is somewhat surprising that most developmental studies so far have focused on the impact of cognitive and monetary incentives on decision-making and goal-directed behavior. In recent years, some researchers suggested that adolescents may be specifically sensitive to perceiving, processing, and responding to social information (e.g., Blakemore and Mills, 2014). In particular, adolescents spend a greater amount of time with peers (Csikszentmihalyi and Larson, 1984) and are increasingly preoccupied with peer opinions (Brown, 1990) and acceptance (Parkhurst and Hopmeyer, 1998). The influence of positive and negative social incentives can be measured in different ways, for instance, by inducing social acceptance/inclusion or social rejection/exclusion from a peer group (e.g., induced with the Cyberball paradigm). Moreover, already the presence of peers or its simulation (e.g., by a chatroom) is sufficient to create a social context that affects decision-making and goal-directed behavior, known as the peer-effect (e.g., Gardner and Steinberg, 2005).

##### Behavioral findings

A number of studies, applying experimental decision-making tasks, have already shown that the presence of peers leads to higher risk taking in adolescents than in adults (Gardner and Steinberg, 2005; Chein et al., 2011; O'Brien et al., 2011; for a review, Albert et al., 2013; Weigard et al., 2014). For instance, researchers have used the so-called Stoplight task, a driving game in which participants advance through several intersections to reach a goal as fast as possible (e.g., Chein et al., 2011). They compared adolescents and two groups of adults in their risky decisions (driving across the stoplight and risking a crash) under conditions in which they performed the simulated driving task either alone or under observation of peers. Only adolescents took more risky decisions under the peer observation compared to the alone condition, while this peer effect was not present in the two groups of adults (Chein et al., 2011). The peer effect can also be obtained by the simulated presence of peers in late adolescents (e.g., Weigard et al., 2014). Interestingly, the peer effect disappeared when a slightly older adult is included into the peer group (Silva et al., 2016), in the presence of the mother (Telzer et al., 2015), and in the presence of an unknown adult (Guassi Moreira and Telzer, 2016), suggesting that this effect is highly sensible to the social context. However, one may argue that the Stoplight task is a rather specific risk-taking setting so that the peer effect cannot be generalized to other risk-taking tasks. Two recent studies have used the Balloon Analogue Risk Task (BART; Lejuez et al., 2002) or an adapted version of it in which a simulated balloon can be inflated via a balloon pump (button press). Each pump signifies a small win that can be accumulated within a trial. After each pump, participants have the choice to either save the money, or to inflate the balloon further, taking the risk for the balloon to burst and to lose the already accumulated money. Indeed, both studies were not able to replicate the peer effect when measuring risk taking by the overall number of inflated balloons (Reynolds et al., 2014; Kessler et al., 2017).

The direct reaction of peers, such as including or excluding an individual into the peer group, may be a stronger incentive for adolescents than the sole presence of a peer. Feelings of exclusion and inclusion from a group often have been induced with the so-called Cyberball paradigm (for details, see Williams et al., 2000). It has been shown that the Cyberball task induces distress (Masten et al., 2009; Bolling et al., 2011; Sebastian et al., 2011), threat (Abrams et al., 2011; Sebastian et al., 2011; Van Noordt et al., 2015), and worse mood (Gunther Moor et al., 2012). Evidence for the effects of inclusion/exclusion from a peer group on decision-making and cognitive control are rather scarce so far. Peake et al. (2013) showed that adolescents revealed a tendency for increased GO-decisions in the Stoplight task after being excluded in a preceding Cyberball game. Adolescents with greater susceptibility to peer influence also displayed larger increases in risky decisions after being socially excluded by peers (Peake et al., 2013).

Recently, Jones et al. (2014) investigated whether children, adolescents, and adults learned an association between the probability of receiving positive feedback and a particular peer (feedback stimulus). Unbeknownst to the participants, the probability of receiving a positive incentive from the three peers was experimentally manipulated, with one peer providing incentives rarely (33% of trials), the other frequently (66% of trials), and the last peer on all trials (continuous). Independent of age, rare probability of positive feedback led to a higher error rate than the other two peer conditions. Learning from positive feedback showed a quadratic age trend, with adolescents demonstrating a lower positive learning rate than children and adults. Thus, while children as well as adults reacted faster to peers that were associated with more frequent positive feedback, adolescents seemed to be motivated equally by all positive social incentives.

The reported quadratic age effect has not been found in other learning tasks (Van den Bos et al., 2012; Christakou et al., 2013). Therefore, either adolescents did not learn to discriminate between the peers associated with different amounts of positive social feedback, or the reinforcement learning predictions did not represent the adolescents' behavior. Accordingly, their learning rate profile could be associated with a general higher sensibility for receiving peer approval (Collins and and Steinberg, 2007).

In sum, social-induced feedback, like acceptance and rejection by peers, but also their mere presence, has an impact on adolescent decision-making. When observed by peers, whether they were present during testing and close friends, or simulated and unknown, adolescents show heightened propensities for risky decisions and immediate rewards. However, only the minority of studies compared different age groups and no study included longitudinal data, making it difficult to account for developmental differences in the influence of social incentives on decision-making and cognitive control behavior over the course of adolescence.

##### EEG findings

Only a handful of ERP studies have investigated the influence of social incentives on electrophysiological markers of decision-making and cognitive control in developmental samples. These studies have revealed several important findings. First, they have shown that the mere presence of peers can influence the significance of (negative) feedback as reflected in the size of the FRN. However, peer presence does not uniformly lead to weakened processing of negative as compared to positive, rewarding feedback, but also depends on the specific situational context (Segalowitz et al., 2011; Kessler et al., 2017). Second, social rejection feedback elicits early (as indexed by the FRN) and later (as indexed by the P3b) feedback processing similar to the FRN after cognitive or monetary feedback with the later processes also depending on peer relationship (Kujawa et al., 2014; Gonzalez-Gadea et al., 2016; Kuo et al., 2017). And third, social exclusion as examined in the Cyberball game elicits larger slow-wave activity (Crowley et al., 2010; White et al., 2012) as well as enhanced medial frontal theta oscillations (Van Noordt et al., 2015), both related to the distress this exclusion causes. However, none of these studies actually examined developmental effects by comparing different age groups or analyzing correlations with age.

##### fMRI findings

fMRI findings on decision-making corroborate the above reported behavioral results by demonstrating adolescent-specific neuronal activations when making risky decisions under conditions of peer observation. The study by Chein et al. (2011) found that 14–18 year-olds had significantly stronger activation of reward-related brain areas, like the VS and OFC, during the execution of risky decisions in the Stoplight task when their peers were watching them. Additionally, activity in these brain regions was associated with risky decision-making as indicated by significantly increased activity for GO relative to STOP trials. In contrast, adults showed no such difference as a function of social context. Moreover, they found that adults engaged several lateral PFC areas more strongly than adolescents, indicating enhanced recruitment of cognitive control. This activation pattern, however, was independent of social context, meaning that an immature cognitive control system in adolescents cannot account for peer influences during risky decision-making. Thus, these findings are conceptually in line with the idea of an enhanced reward-seeking motivation in mid-adolescents.

Similarly, Smith et al. (2015) examined adolescents and adults in a card guessing task that included rewarded and non-rewarded trials. Additionally, social context was manipulated by having participants complete the task both alone and while being observed by peers. When observed by peers, adolescents exhibited greater VS activation than adults, but no age-related differences were found when the task was completed alone. These findings suggest that during adolescence, peer presence influences recruitment of reward-related regions in a reward-processing task even when this task involves no risk taking at all.

Concerning the processing of social acceptance and rejection feedback, Gunther Moor et al. (2010) examined children, early adolescents, older adolescents, and young adults in a social judgement task. They presented photographs of peers and asked their participants to predict whether they would be liked by this person. This was followed by feedback (“yes” vs. “no”) indicating whether the person actually liked them or not. Their results showed that the expectation to be liked was accompanied by activation of the ventromedial PFC (a region known to be involved in processing of self-relevant information) and the striatum. This activation was similar in older adolescents and adults, but less pronounced in children and early adolescents. Furthermore, when the expectation to be liked was followed by social acceptance feedback, the ventromedial PFC and the striatum were similarly responsive across all age groups. In contrast, when the expectation to not be liked was followed by negative feedback, the striatum, subcallosal cortex, paracingulate cortex, later PDF and OFC showed linear increases in activation with increasing age. Because this activation was also positively correlated to the resistance to peer influence, the authors interpret this finding as adults being better in regulating the negative feelings that are linked to social rejection. These results are not consistent with the notion of an enhancement of social feedback processing in adolescence. However, they highlight the importance of positive social feedback in general, because already children at the age of 8–10 years were rather sensitive to acceptance feedback.

In contrast, a very similar study by Jones et al. (2014) demonstrated an enhanced sensitivity to unexpected social acceptance feedback in adolescents as compared to children and adults. The authors compared the effects of social reinforcement (receiving a note vs. receiving no note) by peers that differed in the amount of positive reinforcement they gave (rare to frequent). The results showed that especially in adolescents, the anterior to mid insula response was correlated with the positive prediction error (receiving a note from a peer that gave positive reinforcement only rarely). This finding may indicate an enhanced salience of positive social reinforcement during adolescence. Additionally, adolescents activated regions responsible for response planning (putamen and supplementary motor area) more than children and adults when they received positive social reinforcement, which suggests that peer approval may motivate adolescents toward action. VS and medial PFC were equally engaged across age, which could reflect that the perceived value of peers based on their reinforcement history was equivalent for children, adolescents, and adults. These findings suggest that fundamental reinforcement learning mechanisms support social reinforcement learning from late childhood to adulthood. In contrast, the heightened activity in the insular cortex and regions within response planning circuitry of adolescents may suggest an affective-motivational sensitivity toward any peer approval.

Together, the reported fMRI data reveal that risky decisions seem to be rewarding for adolescents because they lead to activation in reward-related neuronal circuitry. Additionally, peer presence enhances the recruitment of these reward-related brain areas and can also dampen activity in a fronto-parietal network responsible for performance in cognitive tasks. As opposed to the studies on peer presence, the results of the few studies examining social acceptance and rejection feedback are less consistent and clearly further research is needed comparing several age groups or even groups differing in pubertal status.

#### Direct comparisons of secondary incentives

Only a small number of studies so far have directly compared the impact of different kinds of incentives on decision-making and goal-directed behavior in adolescence and the underlying neuronal circuitry. These studies are primarily motivated by the claim that different types of incentives share the same neuronal basis, supporting the idea of a “common neural currency” of rewards, and investigating atypical processing of rewards in clinical subsamples that will be not reported here (e.g., Autism Spectrum Disorder, Internet-addicts). We found one study that directly compared primary and secondary incentives that is reported in section Primary Incentives, so that we will focus here on comparisons between secondary incentives. We will also not include studies investigating only adults (e.g., Izuma et al., 2008; Flores et al., 2015), clinical subsamples (e.g., Lin et al., 2012; Kim et al., 2014; Gonzalez-Gadea et al., 2016), or one age group (Op de Macks et al., 2017).

In an attempt to compare social and monetary feedback, Ethridge et al. (2017) tested differential neural responses to social and monetary incentives in young-adolescents and emerging adults. Social feedback was induced through acceptance and rejection feedback while participants engaged in the so-called Doors task (Proudfit, 2014). Positive and negative feedback in the social condition was indicated by a green “thumbs up” for acceptance or by a red “thumbs down” for rejection. In the monetary condition, a green arrow pointing up indicated a win of $0.50 and a red arrow pointing down indicated a loss of $0.25. In addition, adults were informed that they could win up to $10, whereas young-adolescents were informed that they could win only up to $5, while all participants were given in fact $5 following the monetary decision-making task. During feedback presentation, the author reported an enhanced reward positivity for social acceptance and winning money as compared to social and monetary negative feedback. The results revealed that the young adolescents showed this effect on both types of incentives, thus did not differentiate between them. In contrast, the adults showed a larger positivity to monetary than social positive incentives, suggesting at first glance developmental changes in the relative importance of incentive cues. However, the findings are difficult to interpret as adults could win twice as much as adolescents which can also explain the differences between the age groups.

## Discussion

The overarching aim of this review was to provide an overview on the impact of different kinds of incentives, in particular, monetary, cognitive, and socials ones, on age differences in decision-making and cognitive control tasks. We were specifically interested in answering the following questions: (1) Do we find age differences in how different kinds of incentives motivate behavior in these tasks; (2) if so, do these age differences primarily occur during anticipation or consuming/receiving incentives; (3) is there evidence for common or distinct neuronal activations across incentives, as well as for age differences in recruiting incentive-related brain regions.

### Do different kinds of incentives motivate behavior differently during adolescence?

Considering the overall findings at the behavioral level, most of the studies did not find age differences in the impact of different kinds of incentives on decision-making and cognitive control. Although primary incentives are more salient in childhood and adolescence than in adults (based on subjective self-reports), they did not modulate age differences in behavioral choices itself. Monetary as well as cognitive incentives led to better task performance in most studies, but again there were no age-differential effects in these benefits or on behavioral adjustments. Again, adolescents differed from other age groups in self-reported positive feelings about gaining money and there was only few evidence that adolescents were more prone to receiving or not receiving larger monetary incentives. If at all, it seems that social incentives have an age-differential effect on adolescents' decision-making in terms of taking higher risks in the presence, acceptance or rejections of peers. However, only rather few studies compared different age groups and it remains unclear whether the peer effect is restricted to very specific task settings. These findings suggest that different kinds of incentives did not differ in their impact on age differences in decision-making and cognitive control behavior. Only one recent developmental study directly compared monetary and social incentives and found that adults were more responsive to monetary than social ones, while no such difference was obtained for young adolescents. However, given the small number of developmental studies so far, future research directly comparing the differential functions of incentives throughout adolescent development is clearly warranted.

### Do different kinds of incentives influence neuronal mechanisms during anticipating and receiving incentives differently in adolescents?

Although an ERP approach is well suitable to examine cognitive and neuronal mechanisms separately in stages of anticipating and receiving incentives, only rather few developmental studies have made use of it to determine age differences in decision-making and cognitive control. Studies investigating the impact of monetary incentives mostly used reinforcement learning tasks and clearly found that both the magnitude and valence of incentives influence feedback processing, in contrast to the anticipation of incentives (here error processing). There is scarce evidence for gender by age interactions on processing monetary incentives but these findings need to be replicated before strong conclusions can be drawn. Studies investigating the impact of cognitive incentives focused on the examination of receiving incentives and again found no evidence supporting the view that adolescents process cognitive incentives differently from adults. Only children were less able to differentiate between positive and negative cognitive incentives as compared to adolescents, due to their immature cognitive control system.

### Age differences in the recruitment of incentive-related brain regions during anticipating and receiving incentives

In adults, there are first reviews and meta-analytic studies pointing to an overlapping processing network for different types of incentives including the ventral medial PFC, OFC, medial PFC, ACC, the posterior cingulate cortex (PCC), the inferior parietal lobule and some regions of the lateral PFC in decision-making (e.g., Liu et al., 2011; Sescousse et al., 2013). Such overlap in recruitment of incentive-related brain regions was found when comparing the processing of primary and secondary incentives (e.g., Lieberman and Eisenberger, 2009; Bartra et al., 2013; see Sescousse et al., 2013, for a review), as well as when comparing social and non-social decision-making (for reviews, Amodio and Frith, 2006; Ruff and Fehr, 2014; see also Saxe and Haushofer, 2008).

To determine age-related differences between adolescents and adults in recruiting incentive-related processing, Silverman et al. (2015) recently reported results of a meta-analysis including 26 fMRI studies. Although they found overlapping brain activation in the incentive-related network, including major nodes such as the ventral and dorsal striatum, insula and the PCC, suggesting that adolescents activate a similar incentive-related network as adults do, adolescents showed a greater likelihood for activation in a number of these regions. However, they also reported age differences in activating brain regions during anticipation and consumption/receipt of rewards. Adolescents showed a larger activation in the insula, amygdala, and putamen during anticipation and larger amygdala activation during receiving feedback, suggesting a higher sensitivity to salient stimuli. When comparing positive to negative incentives, adolescents showed larger activation in the accumbens, PCC, and OFC. Relative to adults, adolescents showed a reduced activation for negative incentives in the amygdala, OFC, and ACC (Silverman et al., 2015).

Concerning the different types of incentives, as reviewed here, it has been shown that adolescents were particularly sensitive to consuming or not receiving primary incentives as reflected in increased activation in the VS relative to adults. A much larger number of studies investigated the impact of monetary incentives and yielded very mixed results: Age differences in brain activations in reward-related and control-related regions were rather inconsistently found depending on the type of task and stage of processing. Generally, it seems that adolescents were sensitive to a “hot” context, that is, when incentive values are high and the probability of receiving them is rather low, an imbalance between the highly activated reward region and low activated control regions may lead to more impulsive and risky decision-making in adolescence. Moreover, receiving negative cognitive incentives led to higher recruitment of control brain regions as well as to a lower amygdala activation, signaling lower sensitivity to potential negative outcomes in adolescents.

## Conclusions and outlook

Most of the developmental studies included in this review compared only two age groups or investigated a restricted age range in a cross-sectional research design. These limitations make it impossible to evaluate current neurobiological developmental models against each other. Comparing different types of incentives and their impact on age differences in decision-making and cognitive control revealed that the effects were quite similar on the behavioral level and mostly age differences were not observable. These findings seem to conflict with the current theoretical models as well as with the research findings on the neuronal level that often showed higher recruitment of control-related brain regions in adults and higher activation in reward-related brain regions in adolescents. Future research thus needs to better integrate and relate the results of different data levels. We also recommend that future research in this field should make more use of neuroscientific methods in order to directly compare differential functions of primary and secondary incentives in different stages of processing (i.e., preparation, response selection, outcome evaluation). This will help us to determine the relative importance of different kinds of incentives on cognitive and neuronal mechanisms. However, the review of findings also revealed that if monetary incentives were rather high, decision options were unknown, or in a social context (presence of peers), adolescents indeed behaved differently compared to adults (and children), at least in particular tasks. Hence, one challenge for future studies will be to further specify in well-controlled studies, which contextual factors are critical for inducing an imbalance between reward and control networks in adolescents, and to also consider the role of individual differences in the subjective valuation of different kinds of incentives across age.

## Author contributions

All authors contributed to the writing of the review. JK wrote the Introduction and Discussion part. HS summarized research findings on cognitive and monetary incentives. CL summarized the behavioral findings of social incentives and NF the neuroscientific findings of social incentives. All authors provided feedback to the other parts. Tables were created by HS, CL, and NF.

### Conflict of interest statement

The authors declare that the research was conducted in the absence of any commercial or financial relationships that could be construed as a potential conflict of interest. The reviewer DPK and handling Editor declared their shared affiliation.
